# Deep learning combining imaging, dose and clinical data for predicting bowel toxicity after pelvic radiotherapy

**DOI:** 10.1016/j.phro.2025.100710

**Published:** 2025-02-01

**Authors:** Behnaz Elhaminia, Alexandra Gilbert, Andrew Scarsbrook, John Lilley, Ane Appelt, Ali Gooya

**Affiliations:** a Centre for Computational Imaging and Simulation Technologies in Biomedicine (CISTIB), Schools of Computing and Medicine, University of Leeds, Leeds, UK; b Leeds Institute of Medical Research at St James’s University Hospital, University of Leeds, Leeds, UK; c Leeds Cancer Centre, St James’s University Hospital, Leeds, UK; d School of Computing Science, University of Glasgow, Glasgow, UK

**Keywords:** Radiotherapy toxicity, Deep learning, Toxicity prediction, Convolutional network, Attention, Pelvic radiotherapy

## Abstract

**Background and Purpose:**

A comprehensive understanding of radiotherapy toxicity requires analysis of multimodal data. However, it is challenging to develop a model that can analyse both 3D imaging and clinical data simultaneously. In this study, a deep learning model is proposed for simultaneously analysing computed tomography scans, dose distributions, and clinical metadata to predict toxicity, and identify the impact of clinical risk factors and anatomical regions.

**Materials and methods:**

: A deep model based on multiple instance learning with feature-level fusion and attention was developed. The study used a dataset of 313 patients treated with 3D conformal radiation therapy and volumetric modulated arc therapy, with heterogeneous cohorts varying in dose, volume, fractionation, concomitant therapies, and follow-up periods. The dataset included 3D computed tomography scans, planned dose distributions to the bowel cavity, and patient clinical data. The model was trained on patient-reported data on late bowel toxicity.

**Results:**

Results showed that the network can identify potential risk factors and critical anatomical regions. Analysis of clinical data jointly with imaging and dose for bowel urgency and faecal incontinence improved performance (area under receiver operating characteristic curve [AUC] of 88% and 78%, respectively) while best performance for diarrhoea was when analysing clinical features alone (68% AUC).

**Conclusions:**

Results demonstrated that feature-level fusion along with attention enables the network to analyse multimodal data. This method also provides explanations for each input’s contribution to the final result and detects spatial associations of toxicity.

## 1 Introduction

The majority of patients undergoing pelvic radiotherapy experience various side effects after radiotherapy (RT), (mainly bowel, urinary and sexual dysfunction) and report that it affects their quality of life [1], [2]. The damage caused by RT to normal tissues depends on various factors and the correlation between these factors and the risk of late toxicity is not well understood [3], [4], [5]. Developing RT-induced toxicity prediction models is essential in mitigating their consequences. Such models can detect various factors associated with toxicity, helping clinicians personalising treatment and minimising the effect of it [6].

Normal tissue toxicity prediction is a large and active field of research. Traditional models rely on dose-volume histogram (DVH) data, aiming to condense dose-volume information into a numerical expression representing the risk of specific toxicities. They simplify the 3D/4D dose distribution into a 1D representation [7]. To incorporate spatial dose information, various Normal Tissue Complication Probability (NTCP) models have been proposed. Examples include spatial feature methods [8], spatial-scale methodologies [9], and dose surface maps methods [10].

With the emergence of machine learning (ML), many studies have employed ML methodologies for toxicity prediction [11], [12]. However, these models also rely on 1D input data. Deep learning, a subset of ML, has shown significant potential for integrating complex 3D spatial information in toxicity prediction. Most prior studies have used single- or multichannel 3D convolutional neural networks (CNNs) to process dose distributions [13], [14] and incorporate other image modalities like computed tomography (CT) scans [15], [16], [17], magnetic resonance imaging MRI [18], and positron emission tomography PET scans [19].

Some research has employed various combinations of data, such as 1D features from DVH, clinical features, and genomic or radiomics features [20], [21], [22], [23], [24]. Although many studies have used data fusion techniques to combine image data and clinical variables [25], [26], relatively few focus specifically on toxicity prediction by analysing dose data combined with clinical features. Ibragimov et al. [27] used a CNN to analyse 3D dose distributions and clinical features for predicting liver stereotactic body radiation therapy outcomes. Welch et al. [28] developed a pipeline that combines clinical data with dose distribution, CT scans, and contour data to predict locoregional failure. In our previous study, we proposed a CNN approach to predict radiotherapy outcomes [29]. However, it only focused on analysing imaging data and planned dose distributions, discarding the analysis of clinical variables.

In this work, we aimed to enhance and refine the previous model by incorporating clinical data. We proposed an attention-based feature fusion approach to include clinical data and identify the significance of each clinical feature. To achieve a comprehensive evaluation, this work investigated three key aspects: (i) Does including clinical data improve the model’s performance? (ii) Does the significance of input data vary depending on the specific bowel symptom being predicted? (iii) How do different combinations of data affect prediction with different models?

**Table 1:** Candidate clinical features considered for toxicity prediction. .

Feature	Mean(std)/number of patients	Missing data	Description
**Diagnosis**			
Cancer type	anal:94, rectal:74, endometrial:48, cervical:97	0	value in {1, …,4}

**Demographic**			
Age	60 (13)	0	patient age (in years) rounded to the nearest integer.
Gender	female:238, male:75	0	1 for male 2 for female
BMI	28 (5)	59/313	kg/m${}^{2}$ rounded to the nearest integer.
Current smoker	yes:48, no:203	62/313	binary value in {0,1}

**Comorbidities**			
Diabetes	yes:25, no:287	1	binary value in {0,1}
Cardiac	yes:101, no:211	1	binary value in {0,1}
Previous surgery	yes:136, no:176	1	binary value in {0,1}

**Medication intake**			
ACE Inhibitors	yes:38, no:273	2/313	binary value in {0,1}
Statins	yes:54, no:257	2/313	binary value in {0,1}

**Treatment**			
Concurrent chemo	yes:206, no:107	0	binary value in {0,1}
Received surgery	yes:117, no:196	0	binary value in {0,1}
Received VMAT	yes:19, no:294	0	binary value in {0,1}
Time since radiotherapy	2.45 (1.2)	0	years after treatment
Recurrence	yes:47, no:266	0	binary value in {0,1}
Total prescribed dose	44.9 (10.4)	0	total irradiated in Gy

**Dose metrics**			
VBowelBag10 Gy	7.2 (6.3)	10/313	% of bowel bag received 10 Gy dose
VBowelBag20 Gy	11.2(10.3)	10/313	% of bowel bag received 20 Gy dose
VBowelBag30 Gy	7.2 (6.8)	10/313	% of bowel bag received 30 Gy dose
VBowelBag40 Gy	16.8 (14.8)	10/313	% of bowel bag received 40 Gy dose
VBowelBag50 Gy	2.2 (4.1)	10/313	% of bowel bag received 50 Gy dose
VBowelBag60 Gy	0.5 (3.4)	10/313	% of bowel bag received 60 Gy dose

**Abbreviations:** std, standard deviation; BMI, body mass index; ACE, angiotensin-converting enzyme; VMAT, volumetric modulated arc therapy. **Note:** all VBowelBagXGy are converted to equivalent dose in 2 Gy per fraction (EQD2).

## 2 Materials and methods

### 2.1 Dataset

A cross-sectional dataset comprised of 313 patients treated curatively with pelvic radiotherapy for anal, rectal, endometrial, and cervical cancer were used in the study. Patients were recruited during follow-up appointments at least one year after radiotherapy and three bowel issues were evaluated: bowel urgency, diarrhoea, and faecal incontinence. CT scans, 3D dose distributions and contour structure sets for organs in the pelvis were collated. Based on Radiation Therapy Oncology Group(RTOG) guidelines [30], the intestinal cavity structure was contoured as ‘bowel bag’ encompassing both large and small bowel regions for each patient and considered as the organ at risk for all three toxicities included in this study. The National Research Ethics Service Leeds East Committee approved the data collection study following ethical review with reference number 13-YH-0156. Further use of data for the current project was provided by the LeedsCAT research database with reference 19-YH-0300. A total of 22 clinical features relevant to bowel urgency, diarrhoea, and faecal incontinence were selected based on prior research that reported potential connections between radiotherapy and bowel toxicity [31], [32], [33], [34]. Furthermore, an expert in patient-reported outcomes after pelvic RT (Dr Alexandra Gilbert) reviewed and further refined the selection. Features were subdivided into cancer type, demographic, comorbidities, medications, treatment and dose metrics (calculated from dose volume histograms, DVHs). Treatment features included treatment type (3D conformal radiation therapy (3DCRT) vs volumetric modulated arc therapy (VMAT)), concurrent chemotherapy treatment (chemo), surgery, time since radiotherapy, and recurrence status of the tumour. Medication use (statins and ACE inhibitors) at the time of questionnaire completion was also included (please see Supplementary Material A for more information about the dataset). Dose metrics also included the total received dose and relative bowel bag volume. Ten patients with missing dose data were removed from the study. In total, 175 of 240 patients reported bowel urgency, 101 of 305 reported diarrhoea, and 84 of 303 reported faecal incontinence. Details of the clinical features are shown in Table 1.

**Fig. 1:**
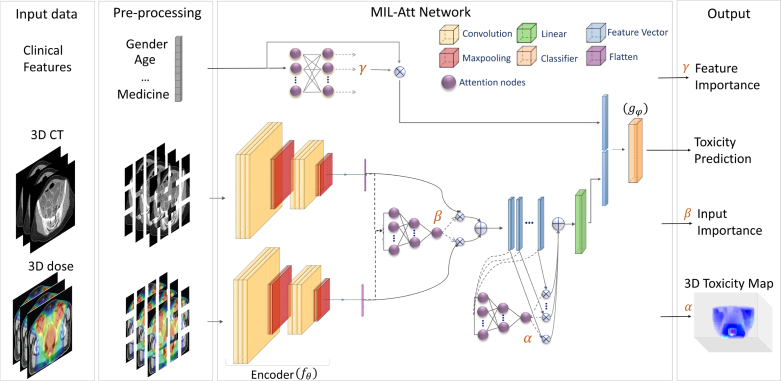
The schematic illustration of the MIL-Att network analysing CT scans, dose distributions, and clinical data. Input data are pre-processed and fed into the MIL-Att network. The output of attention modules is used to detect clinical risk factors, anatomical regions associated with and importance of CT images and dose distributions. The output of the network predicts the toxicity occurrence.

**Fig. 2:**
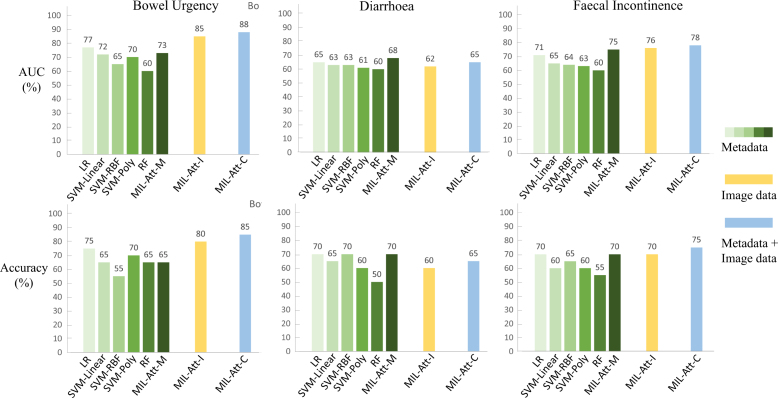
Comparison of prediction performance for various models. Green bars show the different models trained with the same clinical metadata. Abbreviations: AUC: area under the receiver operating characteristic curve; LR: logistic regression; RBF: radial basis function; MIL-Att: multiple instance learning network with attention; MIL-Att-M: network trained with clinical data; MIL-Att-I: network trained on CT and dose data; MIL-Att-C: network trained with combination of clinical data, CT scans and dose distributions.

**Fig. 3:**
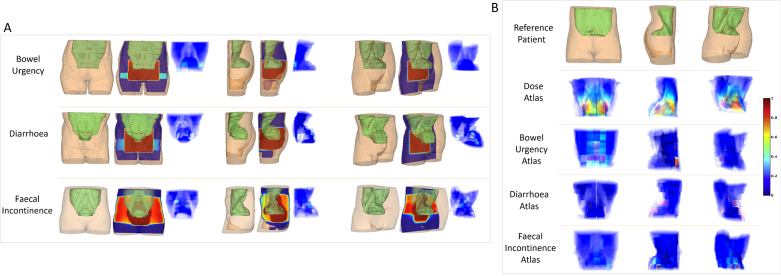
Attention map generated by proposed model. A: example for patient specific attention map; B: the attention atlas generated based on the whole population.

### 2.2 Neural network for multimodal data fusion

We previously presented a two-path network focusing solely on CT imaging and dose distribution data for predicting bowel urgency toxicity [29]. In this work, we expanded and modified our previous network to perform data fusion, enabling simultaneous analysis of clinical data and 3D data. The model already included two attention modules, named α and β. The α attention highlighted the importance of different bowel bag regions, while the β attention indicated the significance of each image data. The higher value of attention weights represented greater importance for the corresponding input. The new architecture incorporated a new attention module and a convolutional block for feature-level fusion. The attention, a fully-connected network, processed 22 clinical features and generated a ranking (γ weights) indicating the impact of each clinical factor on the prediction.

All the images and clinical data were preprocessed for registration and normalisation before being fed to the network (please see Supplementary Material B for details). The clinical features were then weighted by their ranks and concatenated with the image features. To prevent the network from being biased toward the higher-dimensional image features, their size was reduced through a new linear module. A concatenation of weighted features from clinical data and imaging data was used as the final feature for the prediction. Fig. 1 illustrates the general architecture of our model. Detailed explanations of the model formulation, training process and measures to avoiding overfitting are provided in the Supplementary Material C.

**Fig. 4:**
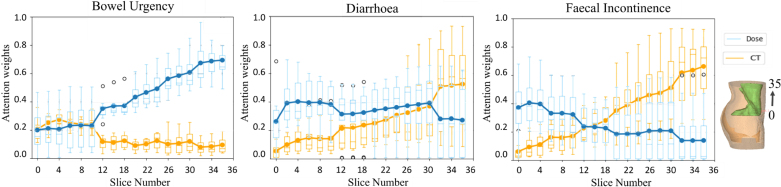
Quantitative evaluation of image association. The y-axis shows the average of the β attention weights for each slice within the bowel bag for both CT and dose distributions. The higher value of attention weight shows higher importance for toxicity prediction. The numbering of slices begins at the caudal end of the bowel bag and increases toward the cranial end.

**Fig. 5:**
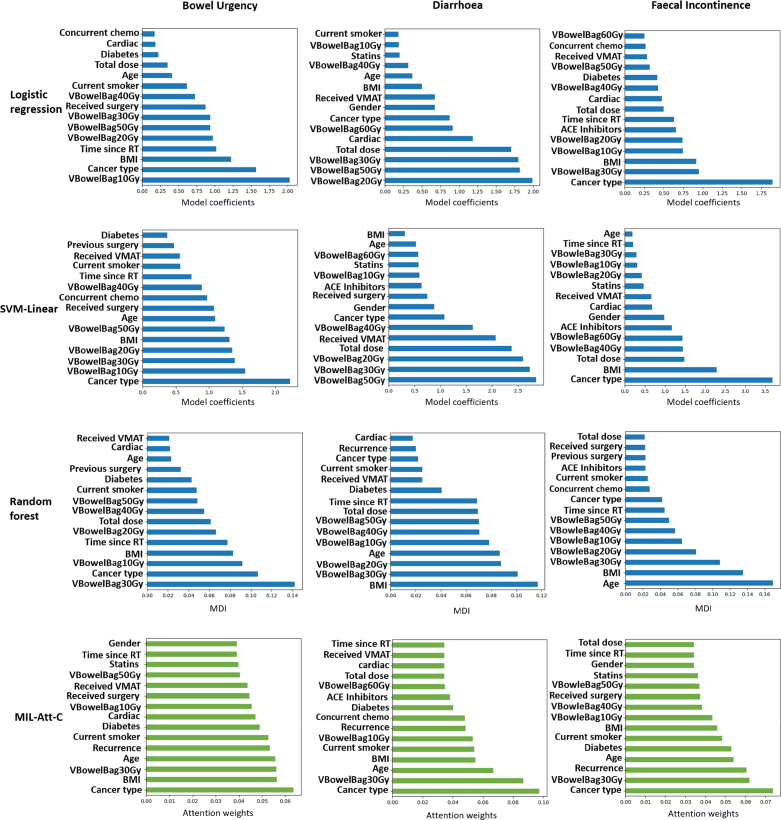
Analysis of risk factors. For clarity, only the top 15 features from each model are presented. The x axis presents the importance of features. The differences in the range of values for feature importance arises from the different computation methods used for each model. **Abbreviations:** BMI, body mass index; VMAT, volumetric modulated arc therapy; RT, radiotherapy; ACE, angiotensin-converting enzyme. MDI, mean decrease of impurity. **Note:** total dose denotes the total prescribed dose..

### 2.3 Analysing different data combination

One of the objectives of this study was to explore how multimodal analysis affects the prediction performance. We evaluated the performance of the proposed model with different input analyses: (i) only clinical features; training model only with the numerical (metadata) data (MIL-Att-M). (ii) spatial features; training model with CT scans and planned dose distributions (image data) only (MIL-Att-I). (iii) both clinical and spatial paths (combined) were trained (MIL-Att-C). Additionally, we analysed three conventional ML models – logistic regression, SVM, and random forest – for toxicity prediction using clinical features, comparing their performance with our proposed model. The SVM used linear, RBF, and polynomial kernels, and the RF model had 100 trees.

We randomly selected 20 patients with and 20 without toxicity for testing, using the rest for training and validation. All models were tested on the unseen test set. To evaluate the network’s prediction performance, accuracy and AUC were evaluated on the test data for all three types of toxicity.

## 3 Results

### 3.1 Toxicity prediction

For bowel urgency and faecal incontinence, jointly analysing CT, planned dose distributions and clinical data improved the accuracy and AUC of the prediction model (see Fig. 2). Analysing only CT scans and planned dose distributions for bowel urgency and faecal incontinence resulted in accuracies of 80% and 70%, respectively. These numbers increased to 85% and 75% when clinical data were added. In contrast, for diarrhoea, training both network paths resulted in a lower AUC than the training clinical features path. The comparison of different models for the same clinical data showed that LR and MIL-Att-M performed slightly better than SVM and RF models.

### 3.2 Analysis of attention maps

Evaluation of the attention weights α revealed that the attention map varied across different toxicities (see Fig. 3.A). Further examination of the atlas for each toxicity indicated that for bowel urgency the weights were concentrated on the inferior and right iliac fossa of the bowel bag. Faecal incontinence was predicted by attention weights in the postero-inferior region (i.e. corresponding to the anorectum), while no clear anatomical region could be identified from the attention weights for prediction of diarrhoea (see Fig. 3.B).

Analysing importance of each CT and dose slice showed that for bowel urgency and diarrhoea, dose slices were more associated, whereas for faecal incontinence, CT slices from the caudal pelvis (slice number < 15) gained more attention (see Fig. 4).

### 3.3 Detecting risk factors (γ weights)

Comparison between the deep learning model and ML models in analysing clinical risk factors showed that for bowel urgency and faecal incontinence, the top ten features had a number of overlapping elements: BMI and cancer type were included almost in the first five important features for all the models. For diarrhoea, dose metrics were prominent for the ML models, while MIL-Att-C only added two to the top 10 list (see Fig. 5).

## 4 Discussion

We proposed an expansion of our previously published study to analyse different combinations of CT imaging, dose distributions, and clinical data to predict bowel-related toxicities. This work has three key novelties and distinctions compared to our previous work. Firstly, the network was expanded and modified to incorporate clinical features for multimodal data fusion. Secondly, a third attention module was employed, to determine the significance of each clinical factor individually. To the best of our knowledge, this study is the first attempt to utilise a deep network for detecting the importance of clinical risk factors in RT toxicity prediction. Lastly, this study analysed three distinct types of bowel toxicity. This more comprehensive evaluation demonstrated the proposed model’s capability to detect different toxicities, reinforcing its reliability in toxicity prediction.

The results showed that the proposed feature-level fusion of CT scan, dose, and clinical data enhanced the prediction performance for both bowel urgency and faecal incontinence. Moreover, incorporating attention modules into the model allowed visual explanations for toxicity distribution and detection of potential risk factors. In particular, the model demonstrated a potential differential spatial dose dependence for different bowel symptoms, likely reflecting diverse underlying pathophysiology.

Before this work, several studies reported deep learning models that incorporate imaging and clinical data for RT outcome prediction [27], [28]. However, the specific significance of each data type in these models remains undetermined, and it is unclear which combination of data is optimal for prediction and why. There are also other studies that compared different combinations of data for deep learning and ML approaches [35], [36], [37], but these studies either cannot detect the data importance or they directly incorporate 3D dose data into the deep learning models.

Analysis of attention module α indicates the anatomical association of toxicity. For bowel urgency, anterior and right iliac fossa regions of the bowel bag were highlighted with a higher possibility of toxicity. The anterior region is the location of most small bowel loops, i.e. representing general irradiation of small bowel. The right iliac fossa region plausibly reflects the dose received to the terminal ileum/caecal region. This area is usually affected in Crohn’s disease and is considered to be involved in bowel symptoms including urgency [38]. This region is not currently considered an avoidance structure in standard radiotherapy procedures, highlighting the need for further investigation in future research. This finding was consistent with our previous work (which considered only CT and dose, without clinical factors [29]). The generated atlas for faecal incontinence showed attention weights focused on the postero-inferior pelvis. From a clinical perspective, this pattern is intuitive and reflects symptom development in relation to anorectal toxicity.

For diarrhoea, the atlas did not demonstrate a clear correlation with a particular anatomical area. This may reflect the fact that diarrhoea is a heterogeneous concept to patients, made up of multiple bowel symptoms such as urgency, stool consistency, frequency and incontinence. These multi-dimensional symptoms may have different underlying mechanisms and therefore may be associated with different parts of the bowel and not a region-specific symptom.

Previous studies have demonstrated the effectiveness of combining different modalities such as CT, MRI or PET [15], [16], [17], [18], but they often lack clarity on how these modalities exert their influence. In contrast, our model with attention module β reveals the significance of each 2D slice. The analysis of β weights revealed varying importance across different slides, indicating distinct impacts of the anatomical structure in CT and spatial information in dose on toxicity prediction. A detailed analysis is in Supplementary Material D.

Incorporating the attention module γ into the network enabled the model to identify which clinical variables were more important for the final prediction, a capability absent in previous studies with similar objectives [27], [28]. The analysis of γ weights and importance coefficients of conventional ML models showed that they more or less selected the same set of features as the top 15 important ones. However, the ML models found dose features more important for the final prediction compared to the MIL-Att network. This suggests that the dose metrics (which do not include spatial information) are generally associated with toxicity, and because the neural network extracts dose metrics separately from dose distributions, the γ weights for these features do not gain high values in the MIL-Att-C model.

Analysis of multimodal data for diarrhoea suggests that the toxicity might be more associated with candidate clinical variables than dose or CT scans. Overall, the network had the lowest accuracy for prediction of diarrhoea symptoms. This may suggest patient-reported diarrhoea describes a more complex issue – one combining stool consistency as well as frequency and urgency – and therefore reflects a more complex aetiology that is dependent on many factors. This will likely be reflected in a less reliable relationship between anatomical dose distribution and the risk of diarrhoea, as reported in this series.

Comparing models trained with clinical data alone, LR and our MIL-Att-M network slightly outperformed other methods, including RF and SVM with different kernels. This might be due to the small dataset and SVM and RF potentially misrepresenting the decision boundary. As probabilistic models, MIL-Att-M and LR performed better, suggesting that clinical features alone determine a probability rather than a definitive outcome.

Several limitations should be noted. First, our dataset had limited size, creating challenges in model generalisation. Ideally, k-fold validation would have been used, but this was not feasible (see Supplementary Material E). Instead, DeLong’s test was conducted for statistical evaluation, with results in Supplementary Material F. Second, there are limitations related to the data itself: some patients underwent brachytherapy, which was not captured in the external beam dose distribution, potentially introducing bias. Future studies should consider both external beam radiotherapy and brachytherapy for a more comprehensive assessment. Additionally, treatments occurred over a decade ago, and the median follow-up period was only two years, factors which may affect the results. Validation with newer datasets and longer follow-up would therefore be beneficial. Furthermore, most patients received 3D conformal radiation therapy (3DCRT) rather than volumetric modulated arc therapy (VMAT). Since newer methods such as VMAT and intensity-modulated radiation therapy (IMRT) have been associated with reduced toxicity compared to 3DCRT, training the model on a more balanced dataset that includes newer treatment techniques could improve generalisation. Finally, the study analysed only the planned dose distributions, which may differ from the delivered dose. For a highly mobile organ like the bowel, spatial dose-dependence may actually reflect spatial variation between planned and delivered doses to specific anatomical substructures. It is also worth noting that the analysis of γ weights showed associations with clinical features but did not clarify the impact of different classes. For example, while BMI was identified as a risk factor, it remains unclear whether a high or low BMI has a greater influence on side effects. Further analysis is needed to address this question.

In conclusion, this study introduced a novel model for multimodal data fusion to predict bowel-related toxicities following pelvic radiotherapy. The proposed framework offered a clinical tool for radiotherapy outcome prediction which can visualise the spatial impact of dose and image information, as well as evaluate clinical risk factors.

## CRediT authorship contribution statement

**Behnaz Elhaminia:** Conceptualization, Methodology, Software, Writing - Original Draft. **Alexandra Gilbert:** Validation, Resources, Data curation, Supervision. **Andrew Scarsbrook:** Validation, Supervision. **John Lilley:** Supervision. **Ane Appelt:** Validation, Data curation, Supervision, Project administration. **Ali Gooya:** Conceptualization, Validation, Supervision, Funding acquisition.

## Declaration of competing interest

The authors declare that they have no known competing financial interests or personal relationships that could have appeared to influence the work reported in this paper.

## Appendix A Supplementary data

MMC S1 Supplementary material on dataset and technical methodology details for deep learning-based bowel toxicity prediction.
