# Supplementary material for: Deep learning combining imaging, dose and clinical data for predicting bowel toxicity after pelvic radiotherapy
Source: Phys Imaging Radiat Oncol. 2025 Feb 1;33:100710. doi: 10.1016/j.phro.2025.100710 (PMC11880715; doi:10.1016/j.phro.2025.100710)
Supplement: MMC S1 — Supplementary material on dataset and technical methodology details for deep learning-based bowel toxicity prediction. [file mmc1.pdf]

Supplementary Material A

Details of Dataset:

A cross-sectional study cohort of patients treated at a single large radiotherapy centre (Leeds Cancer Centre, UK) was evaluated. The dataset comprised 313 patients treated between 2004 and 2014 with pelvic radiotherapy for anal, rectal, endometrial, and cervical cancer. The treatment's intent encompassed all purposes, including radical, palliative, adjuvant, or neoadjuvant. The National Research Ethics Service Leeds East Committee approved the original data collection study (reference 13-YH-0156). Subsequently, further permission for the use of data for the current study was obtained through LeedsCAT research database approval (reference 19-YH- 0300).

Patients were recruited when attending clinical follow-up appointments at least one year after radiotherapy treatment (median two years; interquartile range: 1.4-3.5 years). They completed a validated diagnosis-specific questionnaire based on the European Organisation for Research and Treatment of Cancer (EORTC) Quality of Life questionnaires, with additional questions from the EORTC Item Library on a single occasion [1–5].

For the purpose of this work, three issues were selected for three evaluations:

- (i) bowel urgency ("When you felt the desire to move your bowels, did you have to rush to get to the toilet?"),
- (ii) diarrhoea ("Have you had diarrhoea?"),
- (iii)faecal incontinence ("Have you had leakage of stools from your back passage?").

Item responses were classified into no symptomatic bowel toxicity when participants responded with "not at all" and as having symptomatic bowel toxicity when they responded as "a little", "quite a bit" or "very much". The worst toxicity in the follow-up period was recorded for each patient as late bowel toxicity.

Seven dosimetric features representing the total received dose, and relative bowel bag volume receiving 10, 20, ... 60 Gy effective doses were estimated from differential dose-volume histograms (VBowelBagXGy). Bowel toxicity-relevant comorbidities were defined by three binary features including the presence of diabetes, cardiac history and previous abdominal surgery. Five treatment features included treatment type (3D conformal RT vs volumetric modulated arc therapy [VMAT]), concurrent chemotherapy treatment (chemo), surgery, time since radiotherapy, and recurrence status of the tumour. Medication use (statins and ACE inhibitors) at the time of questionnaire completion was also included. Please note that time since RT and recurrence were added to control for the effects of these factors. This allowed more reliable conclusions to be made about the relationship between other variables and the risk of toxicity.

Summary of treatment

TABLE I: Summary of treatment received for each cancer type

Cancer type	Treatment
Anal	Curative chemoradiotherapy: 50 Gy in 25 fractions EBRT combined with concurrent chemotherapy (mitomycin and 5-flurouracil)
Rectal	Two neoadjuvant (pre-surgery) radiotherapy schedules depending on patient fitness and stage: 25 Gy in 5 fractions EBRT or 45 Gy in 25 fractions of EBRT combined with concurrent chemotherapy (5-flurouracil) followed by curative surgery.
Endometrial	Post surgery patients received EBRT 45 Gy in 25 fractions and some patients received vaginal vault brachytherapy 12 Gy in 3 fractions.
Cervical	Curative chemoradiotherapy: 48 Gy in 24 fractions EBRT combined with concurrent chemotherapy (cisplatin) followed by brachytherapy 21 Gy in 3 fractions.

Abbreviations: Gy, gray(absorbed energy per unit mass of tissue); EBRT, external beam radiotherapy;

Summary of the treatment statistics

TABLE II: Summary of the treatment statistics included in the dataset.

Cancer type	Sex	Num	Age (years)	Total Dose (Gy)	Min dose (Gy)	Max dose (Gy)
Anal	F	67	62.10[9.40]	45.35[7.91]	2.17[4.08]	26.04[18.62]
	M	27	63.95[11.03]	45.478.46]	1.46[3.09]	21.90[19.61]
Rectal	F	26	61.90[17.80]	36.10[11.33]	2.16[1.45]	35.98[11.92]
	M	48	65.15[9.90]	37.0[10.84]	0.37[1.57]	19.23[9.23]
Endometrial	F	48	67.24[11.63]	46.31[5.766]	1.18[3.19]	61.73[17.68]
Cervical	F	97	49.82[12.95]	51.50[8.43]	6.23[7.75]	71.97[12.31]

Abbreviations: Num, number; F, female; M, male. For age, total dose, min dose and max dose, the mean [standard deviation] is shown in the table.

Supplementary Material B

Data Pre-processing:

The values for some data entries in the dataset were not available. The missing value issue causes practical problems for ML models, and they need to be identified and replaced. For categorical values (smoking, etc.) we replaced missing data with the mode. Missing values for continuous data were imputed by multivariate imputation [6], using all other data items as input for the imputation regression model. It is important to note that the regression model used was exclusively trained on the training set. Scikit-learn [7], Python library version 1.1.1 was utilised for all types of statistical imputation. Ten missing entries for dosimetric features (VBowelBagXGy) were not imputed, and we excluded those patients from the study. Continuous features were normalised with Min/Max normalisation to be in range $[0,1]$ and categorical variables were re-scaled with target encoding. Target encoding aims to encode the categories with respect to the impact they might have on the target. For a binary classifier, it computes the posterior probability of target=1, given the input x belongs to the category c_i as: $p(t=1|x=c_i)$. As the output is a value between zero and one, no additional normalisation was required for categorical values.

All dose distributions were converted to equivalent doses in 2 Gy fractions (EQD2, with $\alpha/\beta = 3Gy$) and multiphase plans were converted per phase before summing. Both CT and dose volumes were registered rigidly to a reference image. variations such as patients being in supine or prone positions and having different numbers of slides with varying bowel lengths, the rigid registration was necessary to ensure consistency across the dataset for training the network. The final data had the dimensions of [35, 512, 512] voxels, and were superimposed with the bowel bag contour along with normalisation in a pre-processing stage.

Supplementary Material C
Model Formulation:

We developed a three-path neural network based on multiple instance learning and attention mechanism (MIL-Att-C) to simultaneously analyse CT scans, dose distributions and patient clinical data. We previously presented a network [8] focusing solely on imaging (CT scan and dose distributions) data for predicting bowel urgency toxicity. This study extends and modifies our prior work to be able to combine 1D clinical data alongside 3D imaging and dose treatment information.

The first two paths consisted of convolutional encoders for extracting features from 3D CT scans and 3D dose plans. We employed the concept of multiple instance learning (MIL) to detect critical anatomical regions. Each 3D volume is firstly preprocessed with normalisation and registration and then divided into smaller cubes, and then these cubes are passed through convolutional encoders. Three attention modules are also included to detect the spatial association of toxicity and to assess the influence of each input on toxicity. finally, the concatenation of clinical and image feature vectors is fed to a classification module that generates the output for toxicity. The model is trained by minimising the binary cross-entropy loss function. Detailed information about the network architecture, the mathematics of all the modules, and network training can be found in the supplementary material.

The output of the encoder is prominent features extracted from the input cube. Consider $\mathsf{X}_{i,k}$ as the kth cube from the ith input ($i=1$ for CT and $i=2$ for dose), the feature extraction can be formulated as: $f_{\theta_i}(\mathsf{X}_{i,k}) = \boldsymbol{h}_{i,k}$, where f_{θ_i}, is the encoder -a convolutional network with parameter θ_i- and $\boldsymbol{h}_{i,k}$ is the extracted features.

he first one, attention module β -over input- identifies how two inputs (CT and dose) are involved in toxicity prediction. The attention module is a feed-forward neural network that computes the weights for each feature vector $\boldsymbol{h}_{i,k}$. Attention weights over input can be formulated as:

$$\beta_{i,k} = \frac{exp\{\boldsymbol{w}^T tanh(\mathrm{V}\boldsymbol{h}_{i,k}^T)\}}{\sum_{j=1}^{2} exp\{\boldsymbol{w}^T tanh(\mathrm{V}\boldsymbol{h}_{j,k}^T)\}} \quad (1)$$

where V and $\boldsymbol{w}$ are weights matrix and vector, respectively. Then the total feature vector extracted for the cube k is computed as:

$$\boldsymbol{z}_k = \sum_{i=1}^{2} \beta_{i,k} * \boldsymbol{h}_{i,k}. \quad (2)$$

The second attention module (α) -over space- detects cubes associated with the toxicity. The weights α for cube k can be computed as:

$$\alpha_k = \frac{exp\{\boldsymbol{q}^T tanh(\mathrm{R}\boldsymbol{z}_k^T)\}}{\sum_{j=1}^{K} exp\{\boldsymbol{q}^T tanh(\mathrm{R}\boldsymbol{z}_j^T)\}}, \quad (3)$$

where R and $\boldsymbol{q}$ are the weight parameters for a feed-forward network. Higher weights α show more association with the prediction. The ultimate feature vector for image data is computed as follows:

$$\boldsymbol{s}^I = l_\mu(\sum_{k=1}^{K} \alpha_k * \boldsymbol{z}_k). \quad (4)$$

where l is a fully-connected network (four layers) with parameters μ that aims to reduce the dimension of the extracted features to be the same as numerical data ($\boldsymbol{s}^I \in \mathbb{R}^{1\times 22}$).

Attention over clinical data:
The third path of the network analyses clinical data. 22 clinical features are passed through a fully-connected network (attention module γ). This module computes the importance of each clinical feature regarding the final prediction. Consider $\boldsymbol{h}_3$ as the feature vector presenting clinical data ($\boldsymbol{h}_3 \in \mathbb{R}^{1\times 22}$), the attention weights for clinical feature n can be computed as follows:

$$\gamma_n = \frac{exp\{(\mathrm{M}\boldsymbol{h}_3)_n\}}{\sum_{j=1}^{22} exp\{(\mathrm{M}\boldsymbol{h}_3)_j\}}, \tag{5}$$

where M is the weight matrix of the attention module. Considering $\circ$ as element-wise product, the final feature vector $\boldsymbol{s}^C$ for clinical data is computed as: $\boldsymbol{s}^C = \gamma \circ \boldsymbol{h}_3$.

finally, the concatenation of clinical and image feature vectors is fed to a classification module (g_φ) which is a two-layer fully-connected network with parameter φ. The final output of the network can be written via: $y = g_\varphi(\boldsymbol{s})$, where y is the predicted label for toxicity and $\boldsymbol{s}$ is the concatenation of $\boldsymbol{s}^C$ and $\boldsymbol{s}^I$.

Network training and loss function:
The network can be formulated as Φ_Ω where:

$$\begin{aligned} &y = g_\varphi(\boldsymbol{s}) = \Phi_\Omega(\mathsf{X}), \\ &g_\varphi : \boldsymbol{s} \mapsto [0,1], \quad \Omega = \{\theta, \mu, \varphi, \boldsymbol{w}, \mathrm{V}, \boldsymbol{q}, \mathrm{R}, \mathrm{M}\}. \end{aligned} \tag{6}$$

Considering t as target label for input X, the model is trained by minimizing the binary cross-entropy loss function as:

$$L(t, \Phi_\Omega) = -t log(\Phi_\Omega) - (1-t) log(1-\Phi_\Omega) \tag{7}$$

The loss function is summed over all inputs from the training set and minimization is performed w.r.t. Ω parameters.

All the layers in the network are activated by rectified linear unit (ReLU) function except the last layers of attention modules which employ the Softmax function, and the classification module which uses the sigmoid function.

Avoid Overfitting:

To avoid overfitting, we employed multiple approaches. Firstly, we employed transfer learning; the weights learned from two autoencoders, which were trained only on the training set, were transferred to the proposed network. Secondly, we performed early stopping; during training in each epoch, 40 data were randomly selected to validate the training procedure and monitor the performance of the model on the validation set. The training was stopped once the performance on the validation set started to decrease. Moreover, having an imbalanced dataset, we applied data augmentation to the minority class in the training set. For clinical data, ADASYN Python toolbox [9] was utilised for data augmentation and the class weight property for all methods was set to “imbalanced”. For the augmentation of CT scan and dose data, additive Gaussian noise with a mean of 0 and a standard deviation of 0.1, as well as smoothing recursive Gaussian noise with a sigma of 5 mm across each axis, were randomly applied to the minority class for each toxicity. It is important to highlight that spatial augmentation did not benefit this network due to the attention mechanism’s focus on learning specific instance locations for differentiation. Random translations or rotations disturb these learned

locations, complicating the model's ability to attend to relevant features.

Supplementary Material D

Attention:

Attention α:

The attention module α calculates the significance of each cube in relation to the ultimate prediction. A toxicity map was generated to visually represent the relevant weights of all the cubes within the bowel bags for each patient. This attention module highlights the anatomical areas recognised by the network as being associated with toxicity. The attention maps vary between patients, which reflects the real-world nature of the study cohort encompassing anatomical variation (i.e. size, BMI, gender). To investigate the correlation between anatomical regions and bowel toxicities, an attention atlas was constructed from all the attention maps. For each toxicity, patients with a positive label (patients with toxicity) were selected and their CT imaging was registered to a reference patient using the 3D Diffeomorphic Demons registration algorithm [10]. The patient with the fewest CT slices for the bowel bag structure was selected as the reference patient. Dose distributions and attention maps were also co-registered to the reference patient using the same transformation as their corresponding CT. The average of all the registered attention maps was computed to construct the atlas for each toxicity.

Analysis of Attention β:

The analysis of β weights for bowel urgency showed that both CT and dose were associated for the inferior part of bowel bag, while for the cranial part the dose distribution noticeably gained more weights; the structure of the caudal part of the pelvis differs between patients (as an example, the size and location of the bladder can make a significant difference in the shape of bowel bag inferiorly); the risk of bowel urgency toxicity will rely not merely on the irradiated dose to that region but also on whether the patient has low-lying small bowel loops. Conversely, for the more cranial parts, the bowel shape is less different (only small and large bowel loops in that area) therefore, the received dose becomes the only discriminating factor. In comparison, for diarrhoea, the dose distribution gained slightly higher attention for most of the slices in the bowel bag. This suggests that diarrhoea symptoms will mostly depend on the dose delivered to the bowel. For the last slices (slice number > 30), CT gained slightly higher attention; although with considerable variation across the patient cohort, which potentially indicates that neither dose nor CT are of particular importance in this part of the bowel volume. For faecal incontinence, analysis of β weights suggests that the dose delivered to the lower anorectum area is highly related to the toxicity, with limited dependence on dose to other but for the rest of the regions.

Supplementary Material E

Why cross-validation is not feasible:

(i) for the purpose of transfer learning, we trained an autoencoder on our dataset. Then, we transferred its learned weights to the encoders of a MIL-Att network. In cross-validation, the dataset is divided into *n* folds and, one fold, is selected as the test set. Then in each iteration, the model is evaluated based on that test set. This means that cross-validation tests the model on all the data in the dataset. Now, if we performed cross-validation on our dataset, the data that are in the test fold have been already seen by the autoencoders. This would make the cross-validation results inaccurate. One solution would be to remove the data in the selected fold and train the autoencoder with the rest of the data, then transfer the weights from the autoencoder to the MIL-Att network and train the MIL-Att network. since this process should be repeated in each iteration, it is computationally expensive and not feasible. Instead, we selected 40 patients from the beginning and trained the autoencoders without those 40 patients. We then tested the model on those 40 patients, which were completely unseen data.

(ii) the number of patients with positive labels is generally small in our dataset. If we divide the dataset into different folds for cross-validation, we must have at least one positive label in each fold to be able to evaluate the performance. Considering that we performed data augmentation, it is possible that the positive data in the test fold is an augmented version of real data in the training folds. This evaluation is also not accurate because the data in the test is already in the training set. However, to statistically assess the performance improvement, we conducted the DeLong test. The results are available in supplementary materials.

Supplementary Material F

DeLong's Test:

To assess the significance of performance improvement, we conducted the DeLong test.The combination of the model significantly enhanced predictions for bowel urgency and faecal incontinence in comparison to traditional ML models (p value < 0.05). However, for diarrhoea, the statistical improvement between LR and deep learning models was not significant.

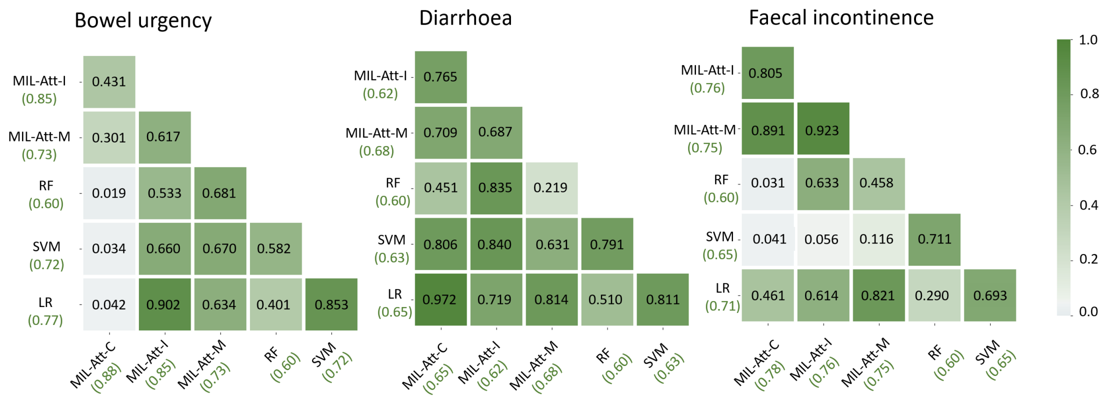

Fig. 1: p value map of DeLong test between the different models in the test sets. AUC values are in parentheses.

References

[1] Aaronson NK, Ahmedzai S, Bergman B, Bullinger M, Cull A, Duez NJ, et al. The European organization for research and treatment of cancer QLQ-C30: a quality-of-life instrument for use in international clinical trials in oncology. J Natl Cancer Inst 1993;85(5):365–376. https://doi:10.1093/jnci/85.5.365.

[2] Greimel ER, Kuljanic Vlasic K, Waldenstrom AC, Duric VM, Jensen PT, Singer S, et al. The European organization for research and treatment of cancer (EORTC) quality-of-life questionnaire cervical cancer module: EORTC QLQ-CX24. Cancer 2006;107(8):1812–1822. https://doi:10.1002/cncr.22217.

[3] Whistance RN, Conroy T, Chie W, Costantini A, Sezer O, Koller M, et al. Clinical and psychometric validation of the EORTC QLQ-CR29 questionnaire module to assess health-related quality of life in patients with colorectal cancer. Eur J Cancer 2009, doi:10.1016/j.ejca.2009.08.014;45(17):3017–3026. https://doi:10.1016/j.ejca.2009.08.014.

[4] Greimel E, Nordin A, Lanceley A, Creutzberg CL, van de Poll-Franse LV, Radisic VB, et al. Psychometric validation of the european organisation for research and treatment of cancer quality of life questionnaire-endometrial cancer module (EORTC QLQ-EN24). Eur J Cancer 2011;47(2):183–190. https://doi:10.1016/j.ejca.2010.08.014.

[5] van Andel G, Bottomley A, Fosså SD, Efficace F, Coens C, Guerif S, et al. An international field study of the EORTC QLQ-PR25: a questionnaire for assessing the health-related quality of life of patients with prostate cancer. Eur J Cancer 2008;44(16):2418–2424. https://doi:10.1016/j.ejca.2008.07.030.

[6] Little RJA Rubin DB. Statistical analysis with missing data. John Wiley & Sons, Inc. 2002. https://doi.org/10.1002/9781119013563.

[7] Pedregosa F, Varoquaux G, Gramfort A, Michel V, Thirion B, Grisel O, et al. Scikit-learn: Machine learning in Python. Journal of Machine Learning Research 2011;12:2825–2830. http://jmlr.org/papers/v12/pedregosa11a.html.

[8] Elhaminia B, Gilbert A, Lilley J, Abdar M, Frangi AF, Scarsbrook A, et al. Toxicity prediction in pelvic radiotherapy using multiple instance learning and cascaded attention layers. EEE J Biomed Health Inform 2023;27(4). https://doi.org/10.1109/JBHI.2023.3238825.

[9] Lemaître G, Nogueira F, Aridas CK. Imbalanced-learn: A Python toolbox to tackle the curse of imbalanced datasets in machine learning. Journal of Machine Learning Research 2017;18(17):1–5. https://www.jmlr.org/papers/v18/16-365.html.

[10] Vercauteren T, Pennec X, Perchant A, Ayache N. Diffeomorphic demons: Efficient non-parametric image registration. NeuroImage 2009;45(1):S61–S72. https://doi.org/10.1016/j.neuroimage.2008.10.040.
